# Strongest chemical weathering in response to the coldest period in Guyuan, Ningxia, China, during 14-11 Ma

**DOI:** 10.1371/journal.pone.0268195

**Published:** 2022-05-05

**Authors:** Qiaoqiao Guo, Hanchao Jiang, Jiawei Fan, Yumei Li, Wei Shi, Siqi Zhang, Xiaotong Wei

**Affiliations:** 1 State Key Laboratory of Earthquake Dynamics, Institute of Geology, China Earthquake Administration, Beijing, PR China; 2 Development Research Center of China Earthquake Administration, Beijing, PR China; Institute of Earth and Environment, Chinese Academy of Sciences, CHINA

## Abstract

Moisture evolution in Central Asia including Northwest China shows less similarity with its surroundings and attracts a growing number of studies. In this study, a well-dated thick lacustrine sequence is chosen in Northwest China and detailed geochemical analysis is conducted during the Middle Miocene Climate Transition (MMCT, 14–11 Ma). The multi-proxy records (Na_2_O/Al_2_O_3_, CIA, Rb/Sr) revealed that chemical weathering was the strongest during 11.85–11 Ma, the coldest period in 14–11 Ma as evidenced by the global deep-sea oxygen isotope records. Accordingly, we conclude that global climate cooled during MMCT and reached the coldest during 11.85–11 Ma. Thus, the westerly circulation became the strongest during this period, which brought more water vapor to Northwest China and the chemical weathering was significantly improved. On the other hand, the significant decrease in temperature led to the marked weakening of evapotranspiration, and thus the effective humidity was relatively increased. Both aspects contribute greatly to the significant enhancement of chemical weathering in eastern Central Asia. This weathering history of the sediments in the northeastern Tibetan Plateau is of great scientific significance to understanding tectonism and climate change in Asia during MMCT.

## 1. Introduction

During the Mid-Miocene Climate Transition (MMCT) period, the earth’s climate experienced long-term cooling, the sea level dropped significantly and the polar ice sheet increased sharply [1–4]. Continental records in Asia show a good response to global cooling during 14–11 Ma, and indicate that global cooling led to not only weakening of the summer monsoon and declining of vegetation cover [5–8], but also strengthening of the winter monsoon as evidenced by coarsening of eolian dust particles [9–11].

Noticeably, recent studies indicate that moisture evolution in Central Asia including Northwest China shows less similarity with its surroundings, mainly expressed as moisture increase with global cooling [11,12]. The topographic change resulting from the Tibetan Plateau uplift was proposed to have strongly influenced the moisture patterns in Central Asia during MMCT [11]. The decrease in sedimentary leaf wax δD_n-alk_ between 15 Ma to 10.4 Ma was used to estimate the gain in elevation ranges between 1.6 km and 2.5 km with a mean of 2.1 km [12]. However, analysis of δ^18^O data from 2750 sedimentary carbonate samples across Asia suggests that a long-standing topographic feature has continuously blocked southerly moisture and subsequent progressive uplift of the Tibetan Plateau has had little impact on the Central Asian climate [13]. Therefore, it remains disputed whether the moisture increase with global cooling during MMCT is attributed to tectonic uplift or the westerlies strengthening.

It is well known that chemical weathering intensity is largely controlled by temperature and precipitation [14,15]. High precipitation and/or warm temperatures can enhance the chemical weathering intensity, whereas either low temperature or decreased precipitation can decrease the chemical weathering intensity [14]. In this study, an analysis of chemical weathering was conducted on the fluviolacustrine sediments to examine the role of westerlies strengthening on the moisture increase in Central Asia during MMCT.

In this study, a well-dated thick lacustrine sequence is chosen in the eastern Liupan Mountains [16]. Given that the Mid-Miocene Climatic Optimum (MMCO) occurred at 16–14 Ma in the Longzhong Basin [17], the detailed geochemical analysis is conducted from 14 to 11 Ma. Our aim is to identify changes in sediment origin and weathering history for the sediments in the northeastern Tibetan Plateau during sedimentation. It is of great scientific significance to understand tectonism and climate change in Asia during the late Middle Miocene.

## 2. Geological and geographical setting

**Fig 1:**
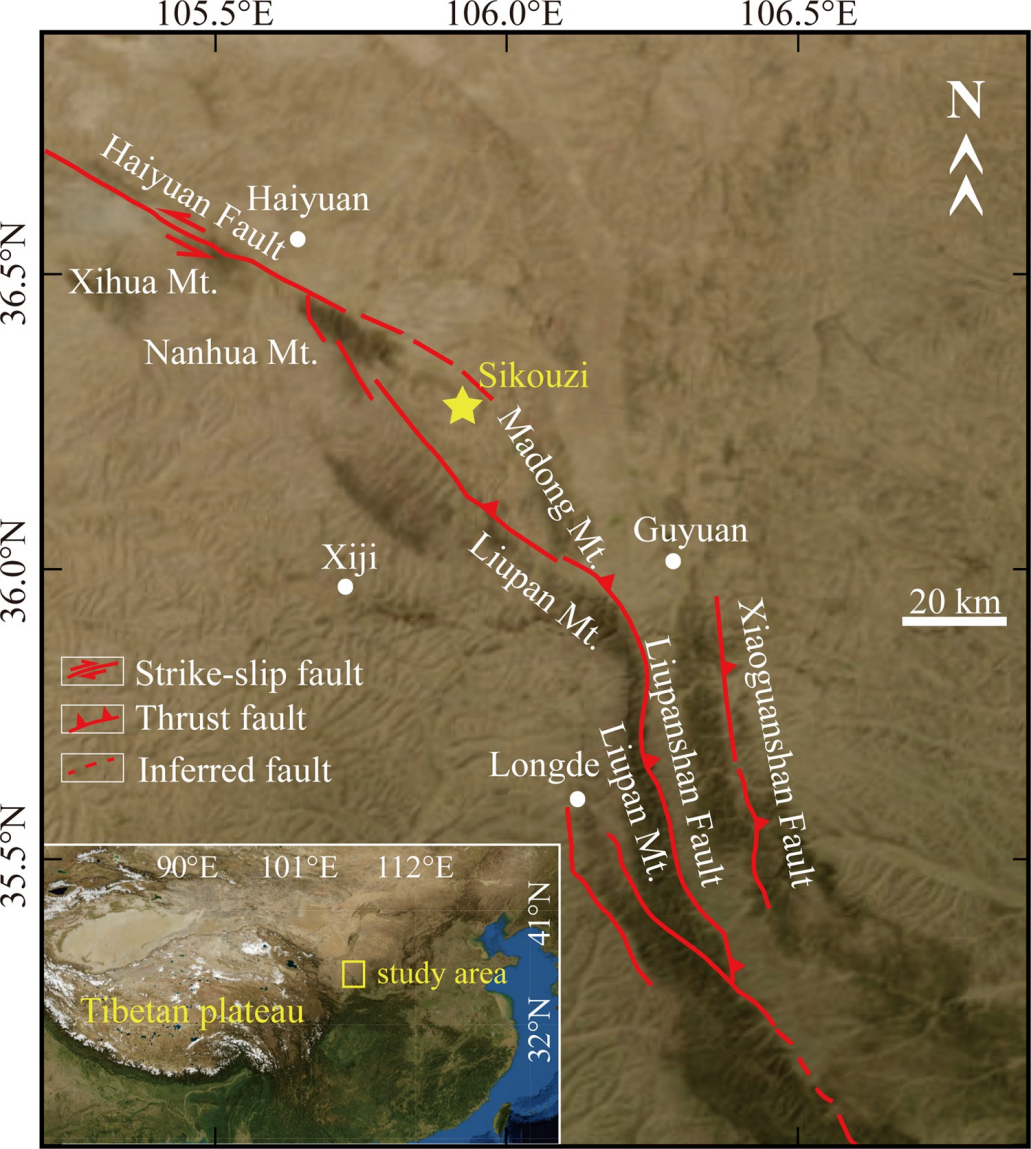
Tectonic setting of Sikouzi Section, Guyuan, Ningxia, China (satellite images download from USGS National Map Viewer (public domain): http://viewer.nationalmap.gov/viewer/; Fault data were from China Earthquake Data Center: http://datashare.igl.earthquake.cn/).

The Sikouzi area is situated on the east side of the Liupan Mountains, approximately 40 km northwest of the town of Guyuan, Ningxia Province, and has a mean elevation of about 1550 m a.s.l (Fig 1). The north of this area is surrounded by the Tengger, Wulanbu, and Mu Us Deserts. Influenced by the East-Asian summer monsoon, climatic conditions of the Sikouzi area at present are temperate and characterized by relatively hot, humid summers and cold winters. For the past 30 years, the mean annual temperature (MAT) is 6.2°C, with a July average of 18.9°C and a January average of -8.3°C. Mean annual precipitation (MAP) is 478 mm, and over 60% of the precipitation falls in July-September with a peak mean rainfall of 109.1mm in August [5,10,18]. Mean annual latent evaporation reaches 1772 mm, which is 3.7 times the annual precipitation. The East Asian winter monsoon, primarily controlled by the Siberian High, drives strong northwestern winds below 2000 m altitude mainly from December to April in this area [5,10,18].

The Sikouzi area is a transitional region between the northeastern margin of the Tibetan Plateau and the Ordos block, which has been relatively stable since the Cenozoic era [19,20]. In the southwest is the Haiyuan-Liupanshan arc fault zone with strong tectonic deformation in the late Cenozoic [20–22], and the Xiangshan-Tianjingshan fault is the boundary in the northeast.

In the study area, reddish and brownish fluviolacustrine sediments, with a thickness of 2880 m, are well exposed along a 5265 m stretch of the Qingshui River. The cross-section map and several photographs of the typical outcrops are presented in Jiang et al. [16]. The lacustrine succession of the Sikouzi area, resting pseudoconformably on Eocene sandstones, strikes NNW and dips ENE with an inclination of 18°-64°. The whole succession includes an anticline and a syncline and is covered unconformably by last glacial loess deposits [16].

Previous studies have suggested that the deformation of the Late Cenozoic strata in this region was caused by the Haiyuan left-slip fault, the recent activity of which began at about 0.2 Ma ago [20,21,23]. The folding caused some difficulty with stratigraphic correlation in the lower part of the section. Fortunately, thick marker beds of white sands in the lower section are distinguishable and the stratigraphic correlation was accurately completed using these marker beds. The lithology of 14–11 Ma is composed of lacustrine shore and deltaic sediments [24], which mainly consist of alterations of greyish and reddish sandstone layers. Thin gypsum layers occur occasionally within the sediments.

## 3. Methods

The exact latitude and longitude (GPS data) of the beginning and end point of the Sikouzi fluviolacustrine section is 36°18′N, 106°02′E and 36°16′N, 105°59′E, respectively. In the field, samples were carried out at intervals of 10–30 m along the Sikouzi section, and there is no need for any permits for field or sample access. Sampling sites were selected where possible in mudstone, silty mudstone, muddy siltstone and siltstone. At each site the surface weathered material was removed and a fresh sample was taken. The samples were stored in the State Key Laboratory of Earthquake Dynamics, Institute of Geology, China Earthquake Administration. Age control for all the samples is derived from a detailed palaeomagnetic record and biostratigraphic data [16]. In this study, thirty samples were selected strictly at an interval of 0.1 Ma spanning 14–11 Ma after age was obtained.

Major and trace element concentrations of bulk samples were determined at the Institute of Geophysical and Geochemical Exploration, Chinese Academy of Geological Sciences following the method of previous studies [25,26]. The samples were fully powdered using an agate mortar, and pretreated to remove organic substances and carbonate components. 10% Hydrogen peroxide was added to remove organic substances. 1 M acetic acid (HOAc) was added to remove carbonate components. Each step was stirred sufficiently, respectively. The solid residues were cleaned by centrifugation three times in pure water. The samples were dried in an oven at 40°C and fully powdered. Approximately 4 g of ground sample was weighed, transferred with boric acid into the center of a column apparatus, and pressurized to 30 t/m2 for 20 s using the ultra-high pressure sample (UHPS) preparation system. The compressed samples, approximately 4 cm in diameter and 8 mm in thickness, were analyzed by PW4400/40 X-ray fluorescence spectrometer. About 0.7 g of ground sample was weighed in a crucible, and put in a muffle furnace to calculate the loss on ignition (LOI).

## 4. Results

**Fig 2:**
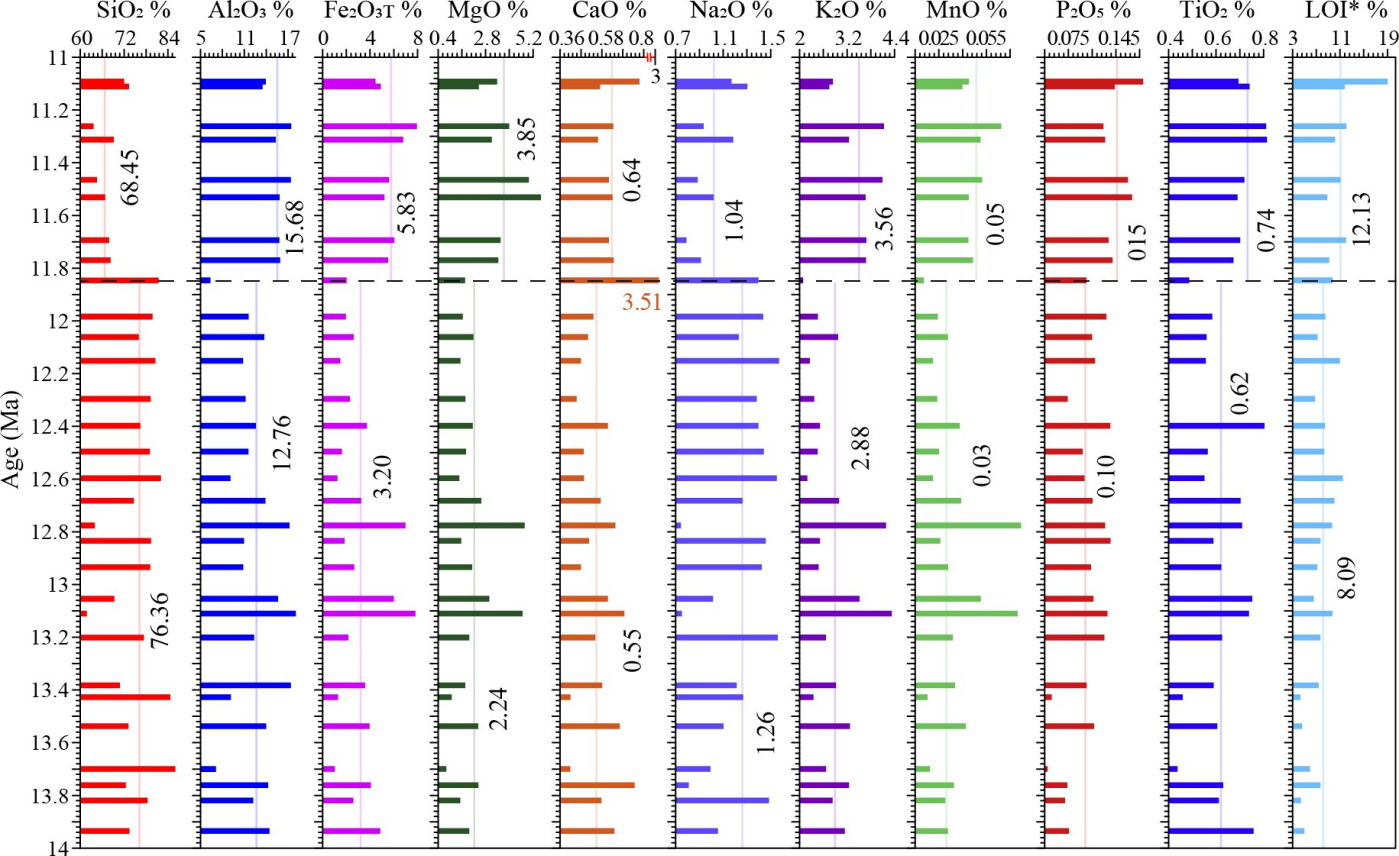
Major element abundances of the fluviolacustrine samples from the Sikouzi section. All major element abundances were recalculated on a volatile-free basis.

The components of major elements of the Sikouzi fluviolacustrine sediments mainly comprise of SiO_2_ (62.09%-86.84%, mean 74.44%), Al_2_O_3_ (6.34%-18.32%, mean 13.32%) and TFe_2_O_3_ (0.73%-7.94%, mean 3.87%) (Fig 2, Table 1). The sum of these three components arrives to 91.63% in total. By contrast, the other major elements are relatively less in abundance, such as K_2_O (2.09%-4.41%, mean 3.04%), MgO (0.82%-5.49%, mean 2.65%), Na_2_O (0.76%-1.59%, mean 1.20%), TiO_2_ (0.43%-0.82%, mean 0.65%) and CaO (0.39%-0.79%, mean 0.58%, except one unusual sample) (Fig 2, Table 1). P_2_O_5_ (0.05%-0.19%, mean 0.12%) and MnO (0.02%-0.08%, mean 0.04%) are even less. Rb varies between 54.4 and 160.3 ppm with a mean value of 100.6 ppm and Sr ranges from 73.4 to 1665.4 ppm with an average of 182.0 ppm (except one unusual sample).

**Table 1:** Major and trace element composition of the fluviolacustrine sediments in Sikouzi section.

Sample	Depth (m)	Age (Ma)^a^	SiO_2_ (%)	Al_2_O_3_ (%)	TFe_2_O_3_ (%)	MgO (%)	CaO* (%)	Na_2_O (%)	K_2_O (%)	MnO (%)	P_2_O_5_ (%)	TiO_2_ (%)	LOI (%)	Rb (μg/g)	Sr (μg/g)	CIA
JXC408	2184.91	11.09	72.23	13.95	4.51	3.47	0.79	1.19	2.91	0.05	0.19	0.71	19.04	96.60	78.50	68.04
JXC409	2189.12	11.11	73.50	13.42	4.89	2.59	0.58	1.32	2.77	0.04	0.15	0.75	12.30	95.60	78.10	68.31
JXC412	2201.76	11.26	63.46	17.76	7.94	4.08	0.65	0.93	4.17	0.07	0.12	0.82	12.40	160.00	73.40	71.02
JXC413	2205.97	11.31	69.36	14.80	6.70	3.15	0.56	1.20	3.23	0.05	0.13	0.82	10.85	132.70	78.00	69.46
JXC416	2218.60	11.46	64.99	17.78	5.69	4.88	0.63	0.90	4.19	0.05	0.16	0.73	11.68	143.00	77.40	71.26
JXC418	2227.02	11.53	66.97	15.95	5.26	5.49	0.66	1.03	3.71	0.05	0.17	0.69	8.84	131.40	78.20	69.73
JXC420	2234.68	11.69	68.33	15.82	6.09	3.68	0.62	0.80	3.77	0.05	0.14	0.71	12.41	130.50	76.30	70.71
JXC421	2238.12	11.77	68.77	15.99	5.56	3.50	0.66	0.91	3.74	0.05	0.14	0.69	9.52	130.00	114.80	70.29
JXC422	2241.57	11.85	81.98	6.34	2.20	1.84	**3.51**	1.41	2.09	0.02	0.10	0.50	10.28	100.60	**16033.10**	36.59
JXC424	2248.46	11.98	79.55	11.58	1.97	1.75	0.53	1.46	2.41	0.02	0.13	0.59	8.95	76.10	394.00	65.93
JXC425	2253.05	12.06	75.86	13.69	2.65	2.32	0.50	1.22	3.03	0.03	0.11	0.57	8.04	86.10	146.50	68.78
JXC427	2262.24	12.15	80.98	10.98	1.50	1.56	0.45	1.59	2.22	0.02	0.12	0.57	11.39	66.90	208.40	65.22
JXC430	2276.02	12.30	79.83	11.23	2.33	1.85	0.43	1.38	2.42	0.02	0.07	0.43	6.78	71.30	97.40	66.42
JXC432	2285.21	12.40	76.12	12.36	3.71	2.25	0.62	1.41	2.56	0.04	0.14	0.80	9.07	94.30	86.50	66.54
JXC433	2289.80	12.50	79.65	11.63	1.65	1.95	0.47	1.46	2.49	0.03	0.10	0.56	8.86	67.40	125.60	66.13
JXC434	2294.39	12.60	82.91	9.37	1.28	1.52	0.47	1.58	2.19	0.02	0.10	0.56	11.83	67.10	1665.40	61.64
JXC435	2298.99	12.68	74.63	13.77	3.27	2.62	0.58	1.27	3.00	0.04	0.11	0.71	10.64	100.30	89.80	68.26
JXC437	2308.17	12.78	63.99	17.52	7.08	4.79	0.66	0.76	4.27	0.08	0.13	0.72	10.20	152.60	77.30	71.22
JXC438	2312.77	12.83	80.56	10.71	1.86	1.66	0.51	1.46	2.49	0.03	0.14	0.59	8.24	77.30	140.80	63.95
JXC441	2326.55	12.93	79.36	10.78	2.71	1.97	0.46	1.45	2.50	0.03	0.11	0.63	7.83	67.90	103.40	64.50
JXC445	2344.92	13.05	69.62	15.35	6.00	2.98	0.62	1.03	3.48	0.05	0.11	0.77	7.20	121.10	78.10	69.94
JXC447	2354.33	13.11	62.09	18.32	7.91	4.86	0.71	0.76	4.41	0.08	0.13	0.75	10.39	160.30	85.10	71.44
JXC450	2368.44	13.20	78.31	11.95	2.19	2.04	0.55	1.56	2.61	0.03	0.13	0.63	8.41	78.40	440.70	65.09
JXC455	2391.96	13.38	71.14	17.82	3.61	1.92	0.58	1.23	2.96	0.03	0.11	0.59	7.84	107.30	308.90	73.90
JXC456	2396.66	13.43	84.35	9.03	1.08	1.09	0.39	1.29	2.25	0.02	0.05	0.46	4.63	54.40	74.80	63.14
JXC458	2406.07	13.54	73.38	14.10	4.00	2.59	0.67	1.12	3.37	0.04	0.12	0.61	5.22	99.50	97.80	67.75
JXC459	2410.78	13.70	86.84	7.12	0.73	0.82	0.39	1.00	2.61	0.02	0.05	0.45	6.03	59.40	103.90	57.91
JXC460	2415.48	13.76	72.77	14.83	4.12	2.62	0.78	0.81	3.32	0.03	0.07	0.64	7.92	108.20	102.90	70.02
JXC461	2420.21	13.82	78.39	11.84	2.62	1.58	0.55	1.50	2.80	0.03	0.07	0.62	4.75	75.90	91.80	64.50
JXC463	2429.68	13.93	73.30	13.92	4.87	2.19	0.64	1.07	3.14	0.03	0.07	0.76	5.61	105.90	104.70	68.73

^a^ Assignment of absolute ages is based on the magnetostratigraphic study [16].

* CaO*: CaO incorporated in the silicate fraction.

^b^ CIA = 100 ⊆ Al_2_O_3_ / (Al_2_O_3_ + K_2_O + Na_2_O + CaO*) [27].

The most significant feature of this record is that the abundance of CaO reaches 3.51% at about 11.85 Ma, which is much higher than the average percentage of 0.58% of the whole sequence during 14–11 Ma. Meanwhile, the percentage of Al_2_O_3_ (6.34%), K_2_O (2.09%) and MnO (0.02%) are the lowest values in the whole record. At the same time, it is noticeable that the Na_2_O/Al_2_O_3_ ratio reaches the maximum value (0.37), CIA arrives at the minimum value (36.6), Rb/Sr runs to the lowest value (0.01) of the whole record. These indicate extremely weak chemical weathering intensity in the study area.

## 5. Discussion

### 5.1 Eolian origin of the Sikouzi fluviolacustrine fine sediments

**Fig 3:**
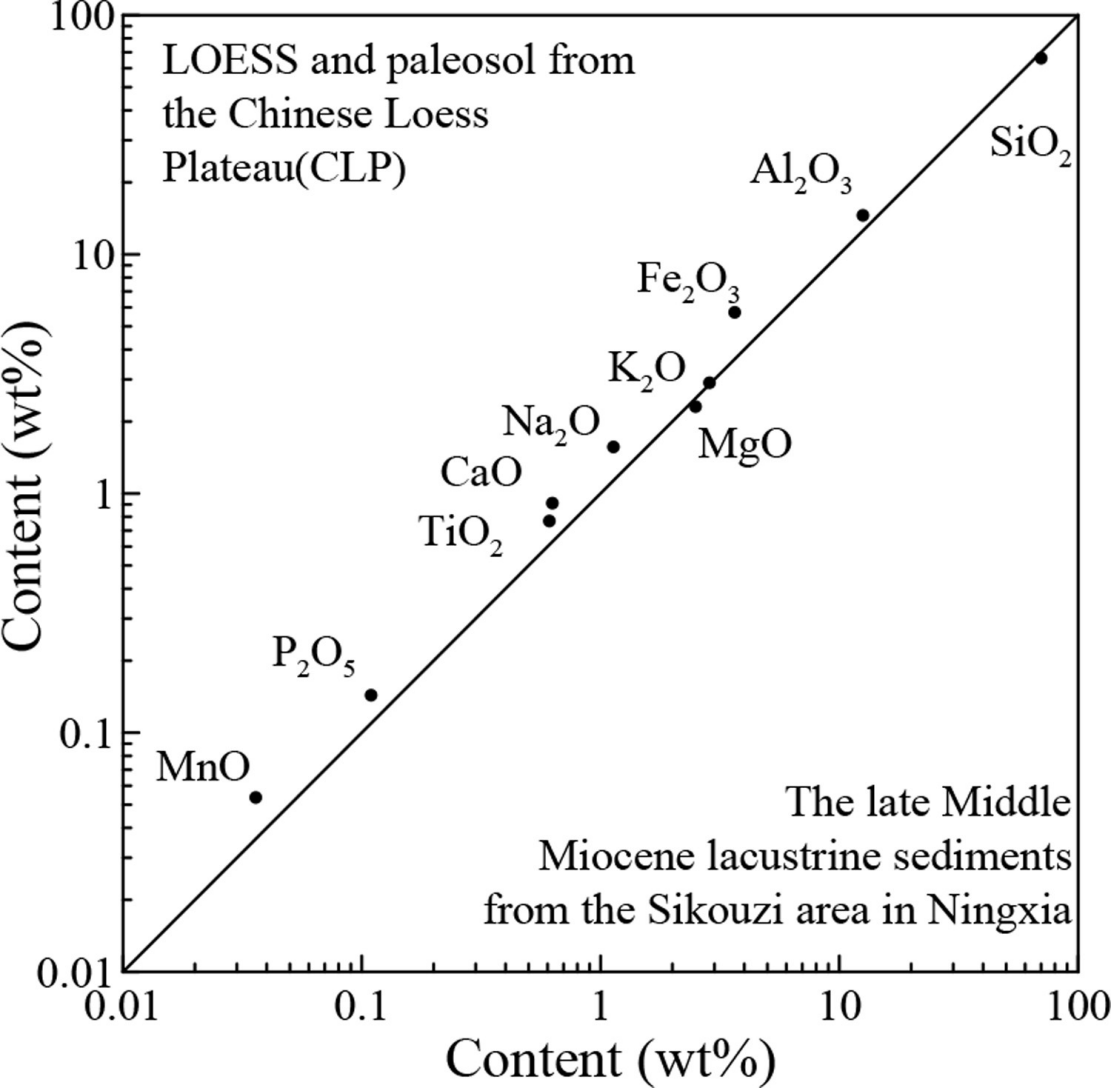
Comparison diagram of major element compositions between the Sikouzi fluviolacustrine fine samples in this study and the loess-soil samples at Baishui in the CLP [28].

It can be seen that all variations for these major elements fluctuate within narrow ranges (Fig 2, Table 1). Such a pattern of the major elements is similar to those observed in the loess-paleosol deposits [28] in the CLP (Fig 3). This good exponential linear correlation suggests that the fine-grained fluviolacustrine sediments from the Sikouzi section are windblown in origin [25,26,29].

**Fig 4:**
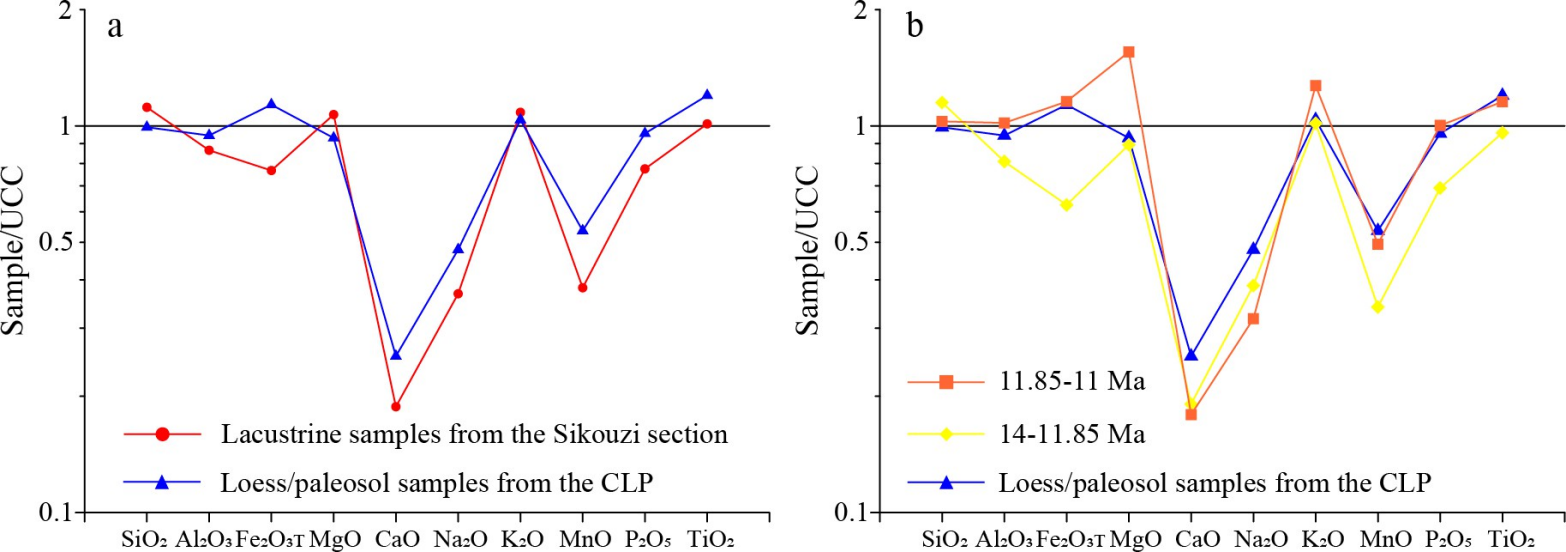
UCC-normalized abundances of major elements for the fluviolacustrine fine samples of the Sikouzi section and for the Baishui samples of loess-soil in the CLP [28]. The UCC values denote Upper Continental Crust compositions from Taylor and McLennan [31] and McLennan [32]. a, all samples; b, two time intervals.

The average chemical composition of the Upper Continental Crust (UCC) can be used to study and compare sediment sources [25,26,30]. The results of Upper Continental Crust (UCC)-normalized abundances for the samples from the study area show a similar pattern to those of typical loess-paleosol sequence in the CLP [28] (Fig 4). This means that the fine-grained sediments in the study area are not only windblown origin, but also possibly have similar dust sources to those of the loess-paleosol sequence in the CLP.

### 5.2 Age model assessment of the late Miocene in the Sikouzi section

Our detailed magnetostratigraphy research was published in 2007 [16]. Subsequently, Wang [22] and Lin [33–35] verified the age model again. After careful comparison, it can be found that the differences in age models for the entire section are very small, and these are even smaller during the late Middle Miocene.

There are several constraints on the comparison of the late Middle Miocene age models. Chron C5n.2n spans about 1 Ma and appears at similar depths in different age models [16,22,33–35]. Tectonic activities occurred in the Sikouzi, Dahonggou and Linxia basin at the almost same time of ~12 Ma [5,36,37] though with a more significant intensity at ~8 Ma around the Liupan Mountains [38–43]. The end-member analysis of all 3499 grain-size samples in Sikouzi lacustrine sediments indicates that the varying trend of three end-members can be successively correlated in seven stages with the integrated benthic δ^18^O record [44]. The magnetostratigraphic record of the Sikouzi section was not only identified the all polarity chron and polar sub-chron but also constrained by the *hipparion* fossils [16]. Furthermore, the enhanced chemical weathering intensity indicated by the multi-proxy records (Na_2_O/Al_2_O_3_, CIA, Rb/Sr) corresponded well in timing with the cooling period revealed by the global deep-sea oxygen isotope records [45] during 14–11 Ma, and there are 9 events of intensified chemical weathering correspond well to the cold periods evidenced by the deep-sea oxygen isotope records [45] within the dating error in this period. (as described below). Based on the above considerations, we still use the previous age model [16].

### 5.3 Geochemical proxies and their environmental implications

The chemical index of alteration (CIA) is often used as a good indicator of the degree of weathering of sediments. It is calculated in molecular proportions as follows: CIA = [Al_2_O_3_/(Al_2_O_3_ + CaO* + Na_2_O + K_2_O] × 100, where the CaO* is the amount of CaO in the silicate minerals [27]. In the process of chemical weathering, Ca, Na and K in feldspar (main silicate) are easy to leach compared with Al. With the enhancement of weathering, the contents of Ca, Na and K in weathering products is reduced, while the contents of Al remained relatively unchanged. Therefore, an increase in the CIA value indicates the enhancement in chemical weathering, and the amelioration of climate [46]. Similarly, the ratio of Na_2_O/Al_2_O_3_ can be used as an indicator of chemical weathering [46,47], as the mother rock undergoes chemical weathering, in which the unstable Na^+^ is leached and the stable cation Al^3+^ is basically unchanged and relatively enriched. Therefore, the smaller Na_2_O/Al_2_O_3_ ratio, the stronger the chemical weathering, and vice versa.

The Rb/Sr ratio is an important indicator of weathering intensity in both lacustrine and loess sediments [48]. In loess sediments, the loss of Sr content is mainly controlled by carbonate loss, Rb may be lost during the transformation of clastic mica and other potassium-bearing minerals into illite and some clay minerals, but the clay minerals can also contain Rb in their structures, so the loss of Rb in paleosoils is much smaller than that of Sr [49]. Therefore, the ratio of Rb/Sr can reflect the strength of chemical weathering of loess strata, high Rb/Sr ratio indicates strong chemical weathering and warm and humid climate while low Rb/Sr ratio indicates weak chemical weathering and cold and dry climate [46,49,50]. Similarly, the high Rb/Sr ratio of lake sediments can reasonably explain the strong chemical weathering in the lake catchment [49,51].

#### 5.3.1 Possible tectonic activity at around 11.85 Ma

**Fig 5:**
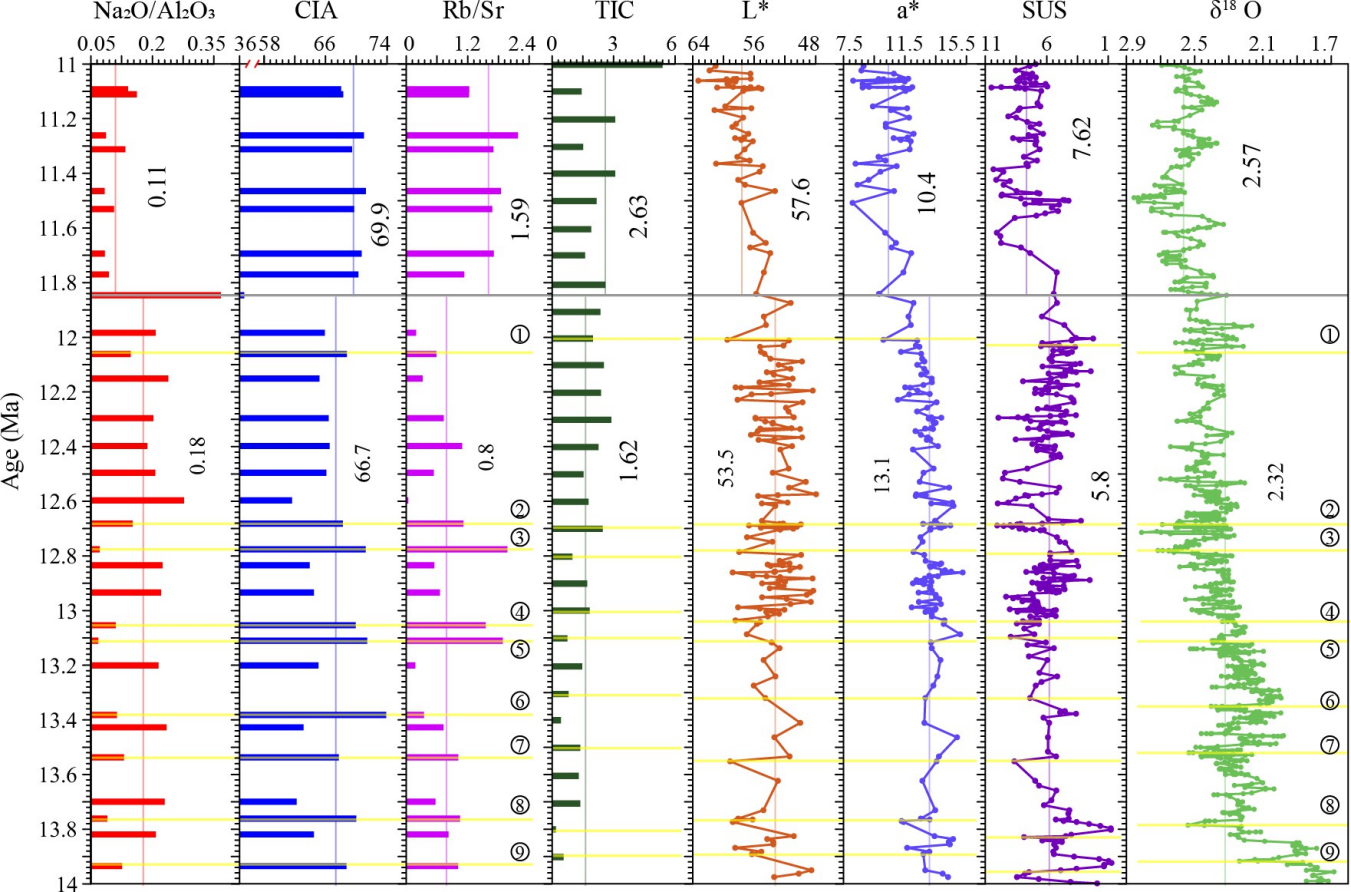
Comparison of variations in Na_2_O/Al_2_O_3_, CIA, and Rb/Sr for the Sikouzi fluviolacustrine sediments with variations in L*, a* [10] and the integrated benthic δ^18^O record [45].

The Sikouzi chemical weathering record can be divided into two parts by 11.85 Ma (Fig 5). There is a significant difference between the upper part (11.85–11 Ma) and the lower part (14–11.85 Ma). From the lower to the upper part, the mean Na_2_O/Al_2_O_3_ ratio decreases from 0.18 to 0.11, CIA increases from 66.7 to 69.9 on average, the mean Rb/Sr ratio increases from 0.8 to 1.59. Comparably, from the Sikouzi sequence, the mean L* value increase from 53.5 to 57.6, the mean a* value decrease from 13.1 to 10.4. What’s more, deep-sea oxygen isotopes increase from 2.32 to 2.57 on average [45].

Intriguingly, the significant change at 11.85 Ma in the study area however has no evident response from the deep-sea oxygen isotope record [45]. On the other hand, several previous studies reveal that tectonic activity occurred widely in the northeastern Tibetan Plateau at the almost same time of around 12 Ma. Carbonate oxygen isotope analysis of lake and river sediments in the Linxia basin shows that, till 12 Ma, the Tibetan plateau has risen high enough to block water vapor from the Indian or South Pacific Ocean from entering western China [36]. Magnetostratigraphic studies of the Dahonggou section in the Qaidam Basin show synchronous tectonic activity of the Qilian Mountains and Altyn Tagh Fault around 12 Ma [37,52]. More records revealing tectonic activity at around 12 Ma in and around the Tibetan Plateau are summarized by Ma and Jiang [53] and in their reference. Accordingly, we consider that a tectonic event occurred around 11.85 Ma in the study area, which can be well correlated and linked with the tectonic change at around 12 Ma in and around the Tibetan Plateau though tectonism occurred more significantly at ~8 Ma around the Liupan Mountains [38–43].

#### 5.3.2 Strongest chemical weathering responds to the coldest period of 11.85–11 Ma

During 11.85–11 Ma, the Na_2_O/Al_2_O_3_ ratio (0.08–0.16) arrives at the lowest mean value (0.11) of the sequence, while CIA (68.04–71.26) and the Rb/Sr ratio (1.13–2.18) reach the highest mean values of 69.85 and 1.59, respectively. These suggest that the 11.85–11 Ma period is marked by the strongest chemical weathering in 14–11 Ma.

Correspondingly, the L* value (53.29–63.27, average 57.59) in 11.85–11 Ma reached the maximum of the whole sequence, indicating the maximum carbonate content [18]. The a* value (8.09–12.06, average 10.42) in 11.85–11 Ma reached the lowest of the Sikouzi sequence, revealing the lowest temperature [18]. These results can be compared with the maximum value of the deep-sea oxygen isotope record [45] during 11.85–11 Ma in the Middle Miocene Climatic Transition (MMCT, 14–11 Ma). This indicates that the chemical weathering in the study area is the strongest when global ice volume reaches the maximum and the temperature reaches the minimum during MMCT.

#### 5.3.3 Several strong chemical weathering events in cold period during 14–11.85 Ma

During 14–11.85 Ma, the Na_2_O/Al_2_O_3_ ratio fluctuates between 0.07 and 0.37 with an average of 0.18, the CIA value varies from 57.91 to 73.90 with a mean value of 66.71, and the Rb/Sr ratio fluctuates between 0.01 and 1.97 with an average of 0.77 (Fig 5). Obviously, the chemical weathering intensity weakens relative to that in 11.85–11 Ma. Noticeably, there are 9 events of intensified chemical weathering in this period, and their intensities are close to that in 11.85–11 Ma. Intriguingly, these 9 events correspond well to the cold periods evidenced by the deep-sea oxygen isotope records within the dating error [45].

### 5.4 Possible mechanism of increased chemical weathering in cold period in eastern Central Asia

High precipitation and/or warm temperatures can enhance the chemical weathering intensity [14]. Considering that 11.85–11 Ma is the coldest period in the whole sequence but with the strongest chemical weathering, we believe that moisture is the most abundant in this period. Generally, there are three hypotheses for the abnormal increase of moisture in Central Asia during global cooling, retreat of the Paratethys, global cooling, and uplift of the Tibetan Plateau.

The proto-Paratethys Sea covered a vast area in Central Asia during the late Eocene and significantly influenced regional climate by providing an immediate source of water vapor [54–56]. However, recent studies show that there were three obvious transgression/regression cycles of the proto-Paratethys Sea [57,58]. The first (from ~59–57 Ma to ~53–52 Ma) and the second (from ~47–46 Ma to ~41–40 Ma) incursion have been poorly constrained, while the third incursion has been precisely dated to 39.7–36.7 Ma. This implies that the final retreat of the Paratethys Sea occurred at 36.7 Ma, and since then, the climate of eastern Central Asia was little affected by the retreat of Paratethys Sea [59].

Uplift of the Tibetan Plateau in the late Cenozoic has generally been believed to have played a significant role in strengthening the Asian monsoon through modulating the atmospheric circulation and its barrier effect to southern-sourced moisture [18]. However, the timing of the uplift of the Tibetan Plateau is controversial. Thermochronometry studies indicate that rapid exhumation of the northeastern Tibetan Plateau started at ~10 Ma in the Qilian Shan, at 8 Ma in the liupan Shan [38–42], at 10–8 Ma along the Kunlun and Haiyuan faults [60] and at ~13 Ma in the Jishi Shan [61]. This uplift timing slices are apparently later than 14–11 Ma. In addition, a compilation of δ^18^O records across Asia suggest that progressive uplift of the Tibetan Plateau has had little impact on Central Asian climate [13].

Global cooling exerts a major effect on changes in water vapor in Central Asia in two aspects. On one hand, global cooling leads to weakening of evaporation and transpiration and consequently increases water vapor content relatively in the study area. On the other hand, global cooling leads to enhancement of westerlies circulation, which transports more Atlantic water vapor to Central Asia. Therefore, we believe that during 14–11 Ma, global cooling led to enhancement of the westerlies circulation, which brought more water vapor into the study area and resulted in the increase of chemical weathering intensity as during 50.5–37.6 Ma in eastern China [47]. This recognation is supported by the improvement of vegetation and climate conditions in the Xunhua Basin during 12.5–8.0 Ma, which was attributed to the decrease in evaporation rates caused by continuous global cooling [62].

## 6. Conclusion

The multi-proxy (Na_2_O/Al_2_O_3_, CIA, Rb/Sr) of the fluviolacustrine sediments at Guyuan, Ningxia, China during MMCT (14–11 Ma) revealed that chemical weathering was the strongest during 11.85–11 Ma. The global deep-sea oxygen isotope records show that temperature during 11.85–11 Ma was the lowest in 14–11 Ma. Accordingly, we conclude that global climate cooled during MMCT and reached the coldest during 11.85–11 Ma. Thus, the Northern Hemisphere climate gradient became the largest and the westerly circulation became the strongest during this period, which brought more water vapor to Northwest China and the chemical weathering was significantly improved. On the other hand, significant decrease in temperature led to the marked weakening of evapotranspiration, and thus the effective humidity was significantly increased. Both aspects contribute greatly to the significant enhancement of chemical weathering in eastern Central Asia.

## References

[1] Shevenell AE, Kennett JP, Lea DW. Middle Miocene Southern Ocean Cooling and Antarctic Cryosphere Expansion. Science 2004; 305: 1766–1770. 10.1126/science.1100061 . 15375266

[2] John CM, Karner GD, Mutti M. δ^18^O and Marion Plateau backstripping: combining two approaches to constrain late middle Miocene eustatic amplitude. Geology 2004; 32(9): 829–832. 10.1130/g20580.1.

[3] Tian J, Yang M, Lyle MW, Wilkens R, Shackford JK. Obliquity and long ec-centricity pacing of the Middle Miocene climate transition. Geochemistry Geophysics Geosystems 2013; 14: 1740–1755. 10.1002/ggge.20108.

[4] Ma XL, Tian J, Ma WT, Li K, Yu JM. Changes of deep Pacific overturning circulation and carbonate chemistry during middle Miocene East Antarctic ice sheet expansion. Earth and Planetary Science Letters 2018; 484: 253–263. 10.1016/j.epsl.2017.12.002.

[5] Jiang HC, Ding ZL. A 20 Ma pollen record of East-Asian summer monsoon evolution from Guyuan, Ningxia, China. Paleogeography Paleoclimatology Paleoecology 2008; 265: 30–38. 10.1016/j.palaeo.2008.04.016.

[6] Miao YF, Fang XM, Herrmann M, Wu FL, Zhang YZ, Liu DL. Miocene pollen record of KC-1 core in the Qaidam Basin, NE Tibetan Plateau and implications for evolution of the East Asian monsoon. Paleogeography, Paleoclimatology, Paleoecology 2011; 299: 30–38. 10.1016/j.palaeo.2010.10.026.

[7] Miao YF, Fang XM, Wu FL, Cai MT, Song CH, Meng QQ, et al. Late Cenozoic continuous aridification in the western Qaidam Basin: evidence from sporopollen records. Climate of the Past 2013; 9: 1863–1877. 10.5194/cp-9-1863-2013.

[8] Jiang H.C., Ding Z.L. Spatial and temporal characteristics of Neogene palynoflora in China and its implication for the spread of steppe vegetation. Journal of Arid Environments 2009; 73, 765–772.

[9] Wan SM, Clift PD, Li AC, Li TG, Yin XB. Geochemical records in the South China Sea: implications for East Asian summer monsoon evolution over the last 20 Ma. Geological Society, London, Special Publications 2010; 342(1): 245–263. 10.1144/SP342.14.

[10] Jiang HC, Ding ZL. Eolian grain-size signature of the Sikouzi lacustrine sediments (Chinese Loess Plateau): Implications for Neogene evolution of the East Asian winter monsoon. Geological Society of America Bulletin 2010; 122(5–6): 843–854. 10.1130/B26583.1.

[11] Miao YF, Herrmann M, Wu FL, Yan XL, Yang SL. What controlled Mid-Late Miocene long-term aridification in Central Asia?—Global cooling or Tibetan Plateau uplift: A review. Earth-Science Reviews 2012; 112: 155–172. 10.1016/j.earscirev.2012.02.003.

[12] Zhuang GS, Brandon MT, Pagani M, Krishnan S. Leaf wax stable isotopes from Northern Tibetan Plateau: Implications for uplift and climate since 15 Ma. Earth and Planetary Science Letters 2014; 390: 186–198. 10.1016/j.epsl.2014.01.003.

[13] Caves JK, Winnick MJ, Graham SA, Sjostrom DJ, Mulch A, Chamberlain CP. Role of the westerlies in Central Asia climate over the Cenozoic. Earth and Planetary Science Letters 2015; 428: 33–43. 10.1016/j.epsl.2015.07.023.

[14] White AF, Blum AE. Effects of climate on chemical weathering in watersheds. Geochimica et Cosmochimica Acta 1995; 59: 1729–1747. 10.1016/0016-7037(95)00078-e.

[15] Berner RA, Berner EK. Silicate weathering and climate. in Tectonic Uplift and Climate Change, edited by Ruddiman W. F. 1997; 353–365, Springer, New York.

[16] Jiang HC, Ding ZL, Xiong SF. Magnetostratigraphy of the Neogene Sikouzi Section at Guyuan, Ningxia, China. Palaeogeography, Palaeoclimatology, Palaeoecology 2007; 243: 223–234. 10.1016/j.palaeo.2006.07.016.

[17] Song Y.G., Wang Q.S., An Z.S., Qiang X.K., Dong J.B., Chang H., et al. Mid-Miocene climatic optimum: clay mineral evidence from the red clay succession, Longzhong Basin, Northern China. Palaeogeography, Palaeoclimatology, Palaeoecology 2018; 512, 46–55.

[18] Jiang HC, Ji J, Gao L, Tang Z, Ding ZL. Cooling-driven climate change at 12–11 Ma: Multiproxy records from a long fluviolacustrine sequence at Guyuan, Ningxia, China. Palaeogeography, Palaeoclimatology, Palaeoecology 2008; 265(1–2): 148–158. 10.1016/j.palaeo.2008.05.006.

[19] Zhang PZ, Burchfiel BC, Molnar P, Zhang WQ, Jiao DC, Deng QD, et al. Late Cenozoic tectonic evolution of the Ningxia-Hui Autonomous Region, China. Geological Society of America Bulletin 1990; 102(11): 1484–1498. 10.1130/0016-7606(1990)102<1484:lcteot>2.3.co;2.

[20] Zhang PZ, Burchfiel BC, Molnar P, Zhang WQ, Jiao DC, Deng QD, et al. Amount and style of late Cenozoic deformation in the liupan shan area, ningxia autonomous region, china. Tectonics 1991; 10(6): 1111–1129. 10.1029/90tc02686.

[21] Burchfiel BC, Zhang PZ, Wang YP, Zhang WQ, Song FM, Deng QD, et al. Geology of the Haiyuan Fault Zone, Ningxia-Hui Autonomous Region, China, and Its Relation to the Evolution of the Northeastern Margin of the Tibetan Plateau. Tectonics 1991;10(6): 1091–1110. 10.1029/90tc02685.

[22] Wang WT, Zhang PZ, Kirby E, Wang LH, Zhang GL, Zheng DW, et al. A revised chronology for Tertiary sedimentation in the Sikouzi basin: Implications for the tectonic evolution of the northeastern corner of the Tibetan Plateau. Tectonophysics 2011; 505: 100–114. 10.1016/j.tecto.2011.04.006.

[23] Burchfiel BC, Deng QD, Molnar P, Royden L, Wang YP, Zhang PZ, et al. Intracrustal detachment within zones of continental deformation. Geology 1989; 17: 448–452. 10.1130/0091-7613(1989)017<0448:idwzoc>2.3.co;2.

[24] Zhang J, Ma ZJ, Ren WJ. The sedimentary characteristics of Cenozoic strata in central and southern Ningxia and their relationships with the development of the Qinghai-Tibetan Plateau. Acta Geologica Sinica 2005; 79(6): 757–773. (in Chinese with English abstract).

[25] Jiang HC, Guo GX, Cai XM, Thompson JA, Xu HY, Zhong N. Geochemical evidence of windblown origin of the Late Cenozoic lacustrine sediments in Beijing and implications for weathering and climate change. Palaeogeography, Palaeoclimatology, Palaeoecology 2016; 446, 32–43.

[26] Liang LJ. and Jiang HC. Geochemical composition of the last deglacial lacustrine sediments in East Tibet and implications for provenance, weathering and earthquake events. Quaternary International 2017; 430, 41–51.

[27] Nesbitt HW, Young GM. Early Proterozoic climates and plate motions inferred from major element chemistry of lutites. Nature 1982; 299: 715–717. 10.1038/299715a0.

[28] Xiong SF, Ding ZL, Zhu YJ, Zhou R, Lu HJ. A ∼6 Ma chemical weathering history, the grain size dependence of chemical weathering intensity, and its implications for provenance change of the Chinese loess-red clay deposit. Quaternary Science Reviews 2010; 29: 1911–1922. 10.1016/j.quascirev.2010.04.009.

[29] Jiang HC, Zhong N, Li YH, Xu HY, Ma XL, Meng YF, Mao X. Magnetostratigraphy and grain size record of the Xijiadian fluviolacustrine sediments in East China and its implied stepwise enhancement of the westerly circulation during the Eocene period. Journal of Geophysical Research-Solid Earth 2014; 119, 7442–7457.

[30] Wang CL, Zhang LC, Dai YP, Lan CY. Geochronological and geochemical constraints on the origin of clastic meta-sedimentary rocks associated with the Yuanjiacun BIF from the Lüliang Complex, North China. Lithos 2015; (212–215): 231–246. 10.1016/j.lithos.2014.11.015.

[31] Taylor SR, Mclennan SM. The Continental Crust: Its composition and evolution. Blackwell, Oxford. 1985.

[32] Mclennan S. Relationships between the trace element composition of sedimentary rocks and upper continental crust. Geochemistry Geophysics Geosystems 2001; 2. 10.1029/2000gc000109.

[33] Lin X, Wyrwoll KH, Chen H. & Cheng X. On the timing and forcing mechanism of a mid-Miocene arid climate transition at the NE margins of the Tibetan Plateau: stratigraphic and sedimentologic evidence from the Sikouzi Section. International Journal of Earth Sciences 2016; 105(3), 1039–1049. 10.1007/s00531-015-1213-z.

[34] Lin X, Chen H, Wyrwoll KH, & Cheng X. Commencing uplift of the Liupan Shan since 9.5Ma: Evidences from the Sikouzi section at its east side. Journal of Asian Earth Sciences 2010; 37(4), 350–360. 10.1016/j.jseaes.2009.09.005.

[35] Lin X, Chen H, Wyrwoll KH, Batt GE, Liao L, & Xiao J. The Uplift History of the Haiyuan-Liupan Shan Region Northeast of the Present Tibetan Plateau: Integrated Constraint from Stratigraphy and Thermochronology. The Journal of Geology 2011; 119(4), 372–393. 10.1086/660190.

[36] Dettman DL, Fang XM, Garzione CN, Li JJ. Uplift-driven climate change at 12 Ma: a long δ^18^O record from the NE margin of the Tibetan plateau. Earth and Planetary Science Letters 2003; 214: 267–277. 10.1016/s0012-821x(03)00383-2.

[37] Lu HJ, Xiong SF. Magnetostratigraphy of the Dahonggou section, northern Qaidam Basin and its bearing on Cenozoic tectonic evolution of the Qilian Shan and Altyn Tagh Fault. Earth and Planetary Science Letters 2009; 288: 539–550. 10.1016/j.epsl.2009.10.016.

[38] Song YG, Fang XM, Li JJ, An ZS, Yang D, Lv LQ. Age of red clay at Chaona section near eastern Liupan Mountain and its tectonic significance. Quat. Sci. 2000; 20, 457–463 (in Chinese with English abstract).

[39] Song YG, Fang XM, Li JJ. The Late Cenozoic uplift of the Liupan Shan, China. Science in China (Series D) 2001a; 44, 176–184.

[40] Song YG, Fang XM, Masayuki T, Naoto I, Li JJ & An ZS. Magnetostratigraphy of late Tertiary sediments from the Chinese Loess Plateau and its paleoclimatic significance. Chinese Science Bulletin 46(Supp.). 2001b; 16–21.

[41] Song YG, Fang XM, Torii M, Ishikawa N, Li JJ, An ZS. Late Neogene rock magnetic record of climatic variation from Chinese eolian sediments related to uplift of the Tibetan Plateau. Journal of Asian Earth Sciences 2007; 30, 324–332.

[42] Zheng DW, Zhang PZ, Wan JL, Yuan DY, Li CY, Yin GM, et al. Rapid exhumation at ∼8Ma on the Liupan Shan thrust fault from apatite fission-track thermochronology: implications for growth of the north-eastern Tibetan Plateau margin. Earth and Planetary Science Letters 2006; 248: 198–208. 10.1016/j.epsl.2006.05.023.

[43] Lu HJ, Malusa MG, Zhang ZY, Guo LC, Shi XH, Ye JC, Sang SP, et al. Syntectonic sediment recycling controls eolian deposition in eastern Asia since ~8 Ma. Geophysical Research Letters 2022; 49, e2021GL096789. 10.1029/2021GL096789.

[44] Jiang H, Wan S, Ma X, Zhong N, & Zhao D. End-member modeling of the grain-size record of Sikouzi fine sediments in Ningxia (China) and implications for temperature control of Neogene evolution of East Asian winter monsoon. PLOS ONE 2017; 12(10), e0186153. 10.1371/journal.pone.0186153. 29023505 PMC5638412

[45] Zachos JC, Dickens GR, Zeebe RE. An early Cenozoic perspective on greenhouse warming and carbon-cycle dynamics. Nature 2008; 451(7176): 279–283. 10.1038/nature06588. 18202643

[46] Ding ZL, Sun JM, Yang SL, Liu DS. Geochemistry of the Pliocene red clay formation in the Chinese Loess Plateau and implications for its origin, source provenance and paleoclimate change. Geochimica et Cosmochimica Acta 2001; 65: 901–913. 10.1016/s0016-7037(00)00571-8.

[47] Jiang H, Zhang J, Zhang S, Zhong N, Wan S, Alsop GI, et al. Tectonic and climatic impacts on environmental evolution in East Asia during the Palaeogene. Geophysical Research Letters 2022; 49, e2021GL096832. 10.1029/2021GL096832.

[48] An FY, Lai ZP, Liu XJ, Fan QS, Wei HC. Abnormal Rb/Sr ratio in lacustrine sediments of Qaidam Basin, NE Qinghai-Tibetan Plateau: A significant role of aeolian dust input. Quaternary International 2018; 469: 44–57. 10.1016/j.quaint.2016.12.050.

[49] Gallet S, Jahn BM, Torii M. Geochemical Characterization of the Luochuan Loess-Paleosol Sequence, China, and Paleoclimatic Implications. Chemical Geology 1996; 133: 67–88. 10.1016/s0009-2541(96)00070-8.

[50] Chen J, An ZS, Wang YJ, Ji JF, Chen Y, Lu HY. Distribution of Rb and Sr in the Luochuan loess- paleosol sequence of China during the last 800 ka. Science in China (Series D) 1999; 42(3): 225–232. 10.1007/bf02878959.

[51] Jin ZD, Li FC, Cao JJ, Wang SM, Yu JM. Geochemistry of Daihai Lake sediments, Inner Mongolia, north China: Implications for provenance, sedimentary sorting, and catchment weathering. Geomorphology 2006; 80: 147–163. 10.1016/j.geomorph.2006.02.006.

[52] Zhuang GS, Hourigan JK, Koch PL, Ritts BD, Kent-Corson, ML. Isotopic constraints on intensified aridity in Central Asia around 12 Ma. Earth and Planetary Science Letters 2011; 312: 152–163. 10.1016/j.epsl.2011.10.005.

[53] Ma XL, Jiang HC. Combined tectonics and climate forcing for the widespread aeolian dust accumulation in the Chinese Loess Plateau since the early late Miocene. International Geology Review 2015; 57(14): 1861–1876. 10.1080/00206814.2015.1027305.

[54] An ZS., Kutzbach JE, Prell WL, Porter SC. Evolution of Asian monsoons and phased uplift of the Himalaya-Tibetan Plateau since late Miocene times. Nature 2001; 411: 62–66. 10.1038/35075035. 11333976

[55] Lu HY, Guo ZT. Evolution of the monsoon and dry climate in East Asia during late Cenozoic: A review. Science China Earth Science 2014; 57: 70–79. 10.1007/s11430-013-4790-3.

[56] Ramstein G, Fluteau F, Besse J, Joussaume S. Effect of orogeny, plate mo-tion and land-sea distribution on Eurasian climate change over the past 30 million years. Nature 1997; 386: 788–795. 10.1007/s11430-013-4790-3.

[57] Kaya MY, Dupont-Nivet G, Proust JN, Roperch P, Bougeois L, Meijer N, et al. Paleogene evolution and demise of the proto-Paratethys Sea in Central Asia (Tarim and Tajik basins): role of intensified tectonic activity at ca. 41 Ma. Basin Research 2019; 31: 461–486. 10.1111/bre.12330.

[58] Meijer N, Dupont-Nivet G, Abels HA, Kaya MY, Licht A, Xiao M, et al. Central Asian moisture modulated by proto-Paratethys Sea incursions since the early Eocene. Earth and Planetary Science Letters 2019; 510: 73–84. 10.1016/j.epsl.2018.12.031.

[59] Ye CC, Yang YB, Fang XM, Zhang WL, Song CH, Yang RS. Paleolake salinity evolution in the Qaidam Basin (NE Tibetan Plateau) between ~42 and 29 Ma: Links to global cooling and Paratethys sea incursions. Sedimentary Geology 2020; 409: 105778. 10.1016/j.sedgeo.2020.105778.

[60] Duvall AR, Clark MK, Kirby E, Farley KA, Craddock WH, Li C. Low tem-perature thermochronometry along the Kunlun and Haiyuanfaults, NE Tibetan plateau: evidence for kinematic change during late-stage orogenesis. Tectonics 2013; 32: 1190–1211. 10.1002/tect.20072.

[61] Lease RO, Burbank DW, Clark MK, Farley KA, Zheng D, Zhang H. Middle Miocene reorganization of deformation along the northeastern Tibetan Plateau. Geology 2011; 39: 359–362. doi: 10.1130/g31356.1

[62] Xu ZL, Zhang JY, Ji JL, Zhang KX. The Mid-Miocene Pollen Record of the Xunhua Basin, NE Tibetan Plateau: Implications for Global Climate Change. Acta Geologica Sinica (English Edition) 2015; 89(5): 1649–1663. doi: 10.1111/1755-6724.12571

